# Validating a short form of the Parental-Caregivers Perceptions Questionnaire (P-CPQ) and the Family Impact Scale (FIS) in Finnish language

**DOI:** 10.1007/s40368-020-00590-2

**Published:** 2020-12-23

**Authors:** A. Keränen, S. Karki, V. Anttonen, M-L. Laitala

**Affiliations:** 1 Research Unit of Oral Health Sciences, University of Oulu, Aapistie 3, POB 5281, 90220 Oulu, Finland; 2 Medical Research Centre Oulu, Oulu University Hospital and University of Oulu, Oulu, Finland

**Keywords:** Children, Oral health-related quality of life, Parents

## Abstract

**Aim:**

This study aimed to adapt the short-form versions of the Parental-Caregivers Perceptions Questionnaire (P-CPQ), and the Family Impact Scale (FIS) in the Finnish language and to test its validity and reliability. Another aim was to compare the background factors of parents with respect to P-CPQ and FIS outcomes.

**Methods:**

This study was conducted among a convenient sample of parents who visited the public dental clinic in Sievi, Finland, from May to October 2016. A total of 54 parents of 2–8-year-old children completed the short-form of the P-CPQ questionnaire and 50 parents of 2–8-year-olds completed the FIS questionnaire while visiting for their children’s routine dental check-up. Parents completed the self-administered P-CPQ and FIS questionnaires. Reliability and validity of the short-form of the P-CPQ and FIS were assessed. Differences between gender, and family size were evaluated using the Mann–Whitney *U* test and the differences between age groups were evaluated using the Kruskal–Wallis one-way ANOVA test.

**Result:**

The Finnish versions of both the short forms of the P-CPQ and FIS had alpha values within the acceptable range. The scales also showed good construct validity. Toddlers (2–4-year olds) had the highest scores for both the P-CPQ and FIS-8 subscales. Likewise, families with 5 or more children had high FIS scores.

**Conclusion:**

The short form of the P-CPQ and FIS in Finnish language are valid and reliable. The oral health of the child seems to have the greatest family impact among parents with five or more children and in families with 2–4-year olds.

## Introduction

Oral health-related quality of life (OHRQoL) measures both functional as well as psychological outcomes of oral diseases (Jokovic et al. 2004). Children with poor oral health status are more likely to experience pain and discomfort resulting in limited activities at school or at home (Jürgensen and Petersen 2009; Seirawan et al. 2012; Karki et al. 2019). Similarly, a recent systematic review concluded that the children with dental pain has a negative impact on OHRQoL (Barasuol et al. 2020). Poor oral conditions may also affect the wellbeing and work of their caregivers and families (BanHani et al. 2018).

The Child Oral Health Quality of Life (COHQoL^®^) includes three sets of age-specific versions of the Child Perceptions Questionnaire (CPQ_6-7_, CPQ_8-10_ and CPQ_11-14_), the Parental-Caregivers Perceptions Questionnaire (P-CPQ), and the Family Impact Scale (FIS). The latter two measures use proxy responses from the parents/caregivers to evaluate the impact of their child’s oral health-related quality of life and the impact of the child’s oral condition on the family (Locker et al. 2002; Jokovic et al. 2003). The P-CPQ and FIS have been translated and validated for different cultural settings (Goursand et al. 2009; Khoun et al. 2018; Kumar et al. 2016). The short-form versions have been found to be reliable and valid (Thomson et al. 2013).

In Finland, all the children and adolescents up to 18 years of age receive free health care including dental health care in Finland. However, children in need of dental treatment are deprived of preventive care compared to those not in need of dental treatment (Linden et al. 2019). The role of parent is crucial in health and well-being of children. Therefore, it is meaningful to evaluate the perception of parents and caregivers with respect to children’s OHRQoL in Finland. Neither the P-CPQ nor the FIS has not been adapted or validated previously in Finland. Therefore, the aim of this study was to adapt the short-form versions of the P-CPQ and FIS in the Finnish language and to test their validity and reliability. Another aim was to compare the background factors reported by parents with P-CPQ and FIS outcomes.

## Methods

### Sampling and study population

This study was conducted among a convenient sample of parents who visited the public dental clinic in Sievi, Finland, from May to October 2016. A total of 54 parents of 2–8 -year-old children (25 boys and 29 girls) completed the P-CPQ questionnaire and 50 parents of 2–8-year olds (20 boys and 30 girls) completed the FIS questionnaire while visiting for their children’s routine dental check-up. All parents were given a chance to respond within a fixed period and no one refused. An information sheet explaining the details of the study was provided to the parents in addition to the P-CPQ and FIS questionnaires. Parents completed the self-administered P-CPQ and FIS questionnaires, while the child was receiving dental examination/treatment.

### Translation and adaptation of the short form of the P-CPQ and FIS

Specific guidelines were followed to produce a cross-culturally adapted Finnish version of the short form of the P-CPQ and FIS (Beaton et al*.* 2000). First, three independent translators translated the English versions of the short forms of the P-CPQ and FIS into Finnish. An expert committee consisting of members of the research group and a dental hygienist working in Sievi developed a second version of both the P-CPQ and FIS incorporating all three translated versions. This version was finally examined by a Finnish linguist reviewer. The same dental hygienist from Sievi pre-tested this final version to confirm the acceptability of the questionnaires.

The responses to the original questions of the P-CPQ were on a four-point Likert scale as follows: ‘Does not bother my child at all’ = 0; ‘Bothers my child a little’ = 1; ‘Bothers my child quite much’ = 2; ‘Bothers my child very much’ = 3 and additionally, a ‘Don’t know’ option was also provided. To be more precise and to avoid possible misinterpretation of the questions, Finnish linguist reviewer suggested to a statement before every question. As suggested by the linguist reviewer, an initial statement on the presence or absence of any oral symptoms during the past 3 months was added to each item of the P-CPQ (Yes/No). After each section, parents had a chance to write comments on the clarity and comprehensibility of the questions.

Concerning the FIS, the response on each item was as follows: ‘Never’ = 0; ‘Once or twice’ = 1;’Sometimes’ = 2;’Often’ = 3;’Every day or almost every day’ = 4, and additionally, a ‘Don’t know’ option was also provided. Parents also had a chance to write comments after each section in the FIS to confirm the clarity and comprehensibility of the questions as in the P-CPQ.

In both the P-CPQ and FIS, background information including the age and gender of the child were also collected. In addition, the opinions of the parents concerning the condition of their child’s oral health and its impact on overall wellbeing were collected. Questions on place of birth (name of the town), and number of children in the family (*1–4 and ≥ 5*) were added in the FIS. The back-translation of the final version did not show any discrepancies except for the responses to the question on their child’s oral health condition. Back-translated responses were *excellent, very good, good, fairly good,* and *poor*. However, in Finnish language, these responses were distinct.

### Research ethics

The primary healthcare centre at Sievi, Finland gave written permission for the study. According to the Finnish legislation, ethical board permission was not necessary on survey-based study (Finlex 1999). Neither the names nor social ID number of either the child or their parents were collected during the survey and study participation was voluntary. Verbal consent obtained from the parents before answering the questionnaires was considered to be consent.

### Statistical analyses

The collected data were transferred into an electronic dataset using IBM SPSS Statistics for Windows, version 24.0 (SPSS Inc., Chicago, USA) for analyses. For analysis, the ‘Don’t know’ option was considered as a score of 0. All the subscale scores in the P-CPQ were computed by summing the discrete subsets of items within each category such as Oral symptoms (four items), Functional limitations (four items), Emotional wellbeing (four items), and Social wellbeing (four items). Similarly, for the FIS, all subscale scores in the P-CPQ were computed by summing discrete the subsets of items within each category such as parental activity (four items), parental emotions (two items), and family conflicts (two items). Finally, the P-CPQ and FIS were computed by summing the scores for all 16 and 8 items, respectively.

To assess the internal consistency reliability of the Finnish short-form of P-CPQ and FIS, Cronbach alphas for the scales and subscales were calculated. Face and content validity were tested. As there was no gold standard, construct validity was evaluated by assessing the correlation between OHRQoL measures (short form of the P-CPQ and FIS total and sub scores) and parent-reported global rating on child’s oral health and impact on overall wellbeing using Spearman Correlation coefficients.

Differences between gender, and family size in the P-CPQ and FIS total and subscores were evaluated using the Mann–Whitney U test and the difference between age groups (2–4-year olds, 5-year olds, and 6–8-year olds) in the P-CPQ and FIS total and subscores were evaluated using the Kruskal–Wallis one-way ANOVA test. For all analyses, a value of *p* ≤ 0.05 was considered statistically significant.

## Results

The mean age of participants for both the P-CPQ and FIS was 5.0 years. A majority of the families had two children, the maximum number of children being 12. The mean (SD) P-CPQ item score was 1.7 (2.17), and mean FIS score was 2.2 (0.65). When comparing the mean values of subscales, the subscale concerning emotions were high in both the P-CPQ (1.13) and FIS (2.3).

The internal consistency reliability showed that the overall P-CPQ had an acceptable alpha value (0.64), whereas for subscales of the P-CPQ, only emotional wellbeing had an acceptable alpha value. Similarly, the FIS and its subscales had acceptable alpha values (Table 1). Concerning the validity, expert reviews and pre-test conducted by the dental hygienist ensured the face and content validity. The parent-reported global rating on child’s oral health was correlated with the oral symptoms subscale of the P-CPQ, whereas the same was true for all other FIS subscales except for the parental conflicts. The impact on overall wellbeing had no significant correlation with either the P-CPQ or FIS (Table 2).

**Table 1:** Mean (SD) values of short form of P-CPQ and FIS subscales separately for boys and girls and Cronbach alphas for the scales

	Total	Boys	Girls	*p*-value	Alpha
P-CPQ respondents (*n*)	54	24	29		
Oral symptoms	0.44 (0.69)	0.32 (0.56)	0.55 (0.78)	0.269	0.05
Functional limitations	0.17 (0.47)	0.04 (0.21)	0.28 (0.59)	0.084	0.35
Emotional well-being	1.13 (1.67)	1.38 (2.16)	0.97 (1.15)	0.907	0.74
Social well-being	0.02 (0.14)	–	0.03 (0.19)	0.363	n.a
Mean PCPQ-16 items	1.7 (2.17)	1.7 (2.38)	1.8 (2.04)	0.534	0.64
FIS respondents (*n*)	50	20	30		
Parental/family activity	2.2 (0.76)	2.24 (0.66)	2.21 (0.83)	0.968	0.703
Parental emotions	2.3 (0.84)	2.33 (0.88)	2.32 (0.83)	0.927	0.841
Family conflicts	2.1 (0.82)	2.24 (0.87)	1.98 (0.79)	0.338	0.731
Mean FIS-8 items	2.2 (0.65)	2.25 (0.55)	2.19 (0.71)	0.751	0.764

*p* values based on Mann–Whitney *U* test

**Table 2:** Parent-reported oral health and its impact on daily performances in association to short form of P-CPQ and FIS (Spearman correlation)

		Global rating on child’s oral health		Impact on overall wellbeing	
		*r*	*p*-value	*r*	*p*-value
P-CPQ	Oral symptoms	0.35	0.009	0.20	0.140
	Functional limitations	− 0.02	0.891	0.20	0.144
	Emotional well-being	0.04	0.804	0.14	0.311
	Social well-being	− 0.10	0.486	− 0.05	0.740
	16 items	0.12	0.369	0.25	0.068
FIS	Parental/family activity	0.271	0.057	0.056	0.704
	Parental emotions	0.243	0.089	0.066	0.654
	Family conflicts	0.045	0.769	− 0.043	0.784
	8 items	0.256	0.072	0.047	0.751

Toddlers (2–4-year olds) had the highest scores for both the P-CPQ and FIS subscales (Table 3). Likewise, families with 5 or more children had a high FIS score. The same was true for the parental emotions subscale (Table 4).

**Table 3:** Mean (SD) values of short form of P-CPQ and FIS subscales separately for different age groups

	Toddlers	5-year-olds	Schoolchildren	*p*
P-CPQ respondents (*n*)	*n* = 17	*n* = 19	*n* = 18	
Oral symptoms	0.6 (0.85)	0.3 (0.58)	0.4 (0.61)	0.407
Functional limitations	0.4 (0.61)	0.1 (0.23)	0.1 (0.49)	0.137
Emotional well-being	1.3 (1.78)	1.2 (2.04)	0.8 (1.02)	0.652
Social well-being	0.1 (0.24)	–	–	0.343
Mean PCPQ-16 items	2.3 (2.38)	1.6 (2.39)	1.3 (1.64)	0.329
FIS respondents (*n*)	14	19	17	
Parental/family activity	2.6 (0.52)	2.0 (0.72)	2.1 (0.86)	0.050
Parental emotions	2.7 (0.46)	2.2 (0.99)	2.1 (0.84)	0.147
Family conflicts	2.3 (0.75)	2.2 (0.86)	1.8 (0.77)	0.128
Mean FIS-8 items	2.6 (0.30)	2.1 (0.72)	2.0 (0.67)	0.031

*P*-values based on Kruskal–Wallis

**Table 4:** Mean (SD) values of short form of FIS subscales separately for different family sizes

	1–4	≥ 5	*p*
	*n* = 24	*n* = 25	
Parental/family activity	2.2 (0.84)	2.3 (0.65)	0.344
Parental emotions	2.1 (0.79)	2.6 (0.85)	0.025
Family conflicts	2.2 (0.75)	2.0 (0.87)	0.449
Mean FIS-8 items	2.1 (0.70)	2.3 (0.57)	0.245

*p* values based on Mann–Whitney *U* test

## Discussion

This study examined the validity of a Finnish short-form of the P-CPQ and FIS among preschool children. The Finnish versions of the P-CPQ and FIS scales appeared to be valid and reliable for evaluating the impact of children’s oral health-related quality of life on families. Parents with five or more children felt sad and worried or guilty more often due to the poor oral health of their child.

The strength of this study was that the validation process was conducted accordingly, and specific guidelines were followed to achieve culturally acceptable and understandable P-CPQ and FIS (Beaton et al. 2000). In addition, parents were also given a chance to comment on the clarity of questions after each section and good feedback was received during pre-testing. However, the sample size was not determined (54 for the P-CPQ and 50 for FIS). This study included the parents of 2–8-year olds in both the P-CPQ and FIS, though it can be used for children of 2–14 years (Zaror et al. 2019). Nevertheless, studies have not defined any particular sufficient sample size for validation of psychometric instruments (Anthoine et al. 2014). Another shortcoming was that we did not perform the test re-rest of the Finnish version of the short form of P-CPQ and FIS.

Children Oral Health-Related Quality of Life (COHQoL^®^) is a well-known questionnaire package comprised of the Parental-Caregiver Perceptions Questionnaire (P-CPQ), the Family Impact Scale (FIS), and three age-specific questionnaires: CPQ_6-7_, CPQ_8-10_ and CPQ_11-14_. In Finland, the CPQ_11-14_ was previously validated and has been used to investigate the OHRQoL among children with and without dental fear (Luoto et al. 2009) as well as children with orofacial cleft (Kortelainen et al. 2016). A recent systematic review concluded that both the P-CPQ and FIS are excellent instruments for measuring the oral health-related quality of life of pre-schoolers, schoolchildren, and adolescents (Zaror et al. 2019). A recent systematic review also suggests using the P-CPQ and FIS among pre-schoolers (Culler et al. 2020). These results justify our decision to validate the short-form of the P-CPQ and FIS to have an instrument to measure family burden and to have a parental perspective as a supplement to children’s self-reported oral health and wellbeing (CPQ). The incorporation of this validated instrument with clinical data can be of value to demonstrate a clear picture of the impact of oral diseases and disorders on the children themselves and their families in Finland.

The Finnish P-CPQ was modified as recommended by an expert during the translation and adaptation process. To make answering more unambiguous, an extra question was added to investigate if the child had any oral symptoms during the past 3 months (yes/no) as suggested by the Finnish linguistic reviewer. The P-CPQ was adapted to ensure the acceptability of Finnish speaking parents. Furthermore, this made the measures clear to the participants as those only with positive answer needs to respond further. In the analyses, a “no” response and ‘don’t know’ were scored as ‘not at all/zero’. This may have skewed distribution of P-CPQ and subscales.

According to our results, the P-CPQ and FIS are reliable in the Finnish context. The P-CPQ and the FIS (including its subscale) showed an acceptable internal consistency here. The results are in full concordance and comparable with the previous literature (Thomson et al. 2013; Goursand et al. 2009). The subscales of the P-CPQ seem to be questionable with respect to reliability except for emotional wellbeing. The emotional well-being subscale of P-CPQ was within the acceptable alpha value. Concerning the construct validity, short form of the P-CPQ and FIS total and sub scores correlated with parent-reported global rating on child’s oral health and impact on overall wellbeing despite being statistical significance.

Parents seem to be more sensitive with respect to emotional well-being as the mean value for this subscale was found to be highest. Similar finding was reported by a recent Brazilian study (Dias et al. 2020). Furthermore, the mean values of the P-CPQ subscales are low (all close to one showing no impact on them). The reason may be that participants in this study were on their routine follow-up, and oral diseases with symptoms are less likely to occur among them. Children up to 18 years of age receive free health care including dental health care in Finland, thus routine preventive care is obvious. The FIS shows that a child’s oral conditions occasionally influence family activities, create family conflicts as well as impact parental emotions even in families of children without oral problems. The mean values of the FIS are higher in those families with 5 or more children, particularly with respect to parental emotion. It seems that parents are quite often upset and have feeling of guilt. If the validation would have been carried out among children with oral symptoms or orofacial disorders, the mean values may have been higher, and needs future studies.

Regardless of the number of children in a family, activities and conflicts are occasionally influenced by children’s oral health. In this study, an impact on families due to a child’s oral conditions was more present among families with toddlers than in families with older children, as measured by the FIS. The same was true with families who had five or more children compared to families with 1–4 children. The proportion of families with five or more children is about half in the present study population, which is more than average in Finland. According to Statistics Finland, in 2014 the mean number of children per family was 1.84; 43% of families have one child, 51% two or three children, and 5% four or more children (SVT, 2014).

As the present study lacks data on clinical examination, a future study should consider evaluating the oral health status in addition to the proxy measures, to get a complete picture of oral health among Finnish preschool children. None of the subscales the P-CPQ (except for the oral symptoms) and FIS-8 subscales were correlated with parent-reported global oral health rating. However, the rho values showed weak correlation in this study needs careful interpretation.

## Conclusion

In conclusion, the short form of the Finnish P-CPQ and FIS are valid and reliable and may be used in future studies. The oral health of the child seems to have an impact on the family as reported most frequently by parents with five or more children and with 2–4-year olds.
